# Modeling Cardiac Disease Mechanisms Using Induced Pluripotent Stem Cell-Derived Cardiomyocytes: Progress, Promises and Challenges

**DOI:** 10.3390/ijms21124354

**Published:** 2020-06-19

**Authors:** Elvira Immacolata Parrotta, Valeria Lucchino, Luana Scaramuzzino, Stefania Scalise, Giovanni Cuda

**Affiliations:** Department of Experimental and Clinical Medicine, Research Center for Advanced Biochemistry and Molecular Biology, University “Magna Graecia” of Catanzaro, 88100 Loc. Germaneto, Catanzaro, Italy; valeria.lucchino@unicz.it (V.L.); scaramuzzino.luana@unicz.it (L.S.); stefania.scalise@unicz.it (S.S.); cuda@unicz.it (G.C.)

**Keywords:** cardiovascular diseases, human induced pluripotent stem cells, cardiac differentiation, iPSC-derived cardiomyocytes, cardiac disease modeling

## Abstract

Cardiovascular diseases (CVDs) are a class of disorders affecting the heart or blood vessels. Despite progress in clinical research and therapy, CVDs still represent the leading cause of mortality and morbidity worldwide. The hallmarks of cardiac diseases include heart dysfunction and cardiomyocyte death, inflammation, fibrosis, scar tissue, hyperplasia, hypertrophy, and abnormal ventricular remodeling. The loss of cardiomyocytes is an irreversible process that leads to fibrosis and scar formation, which, in turn, induce heart failure with progressive and dramatic consequences. Both genetic and environmental factors pathologically contribute to the development of CVDs, but the precise causes that trigger cardiac diseases and their progression are still largely unknown. The lack of reliable human model systems for such diseases has hampered the unraveling of the underlying molecular mechanisms and cellular processes involved in heart diseases at their initial stage and during their progression. Over the past decade, significant scientific advances in the field of stem cell biology have literally revolutionized the study of human disease in vitro. Remarkably, the possibility to generate disease-relevant cell types from induced pluripotent stem cells (iPSCs) has developed into an unprecedented and powerful opportunity to achieve the long-standing ambition to investigate human diseases at a cellular level, uncovering their molecular mechanisms, and finally to translate bench discoveries into potential new therapeutic strategies. This review provides an update on previous and current research in the field of iPSC-driven cardiovascular disease modeling, with the aim of underlining the potential of stem-cell biology-based approaches in the elucidation of the pathophysiology of these life-threatening diseases.

## 1. Introduction

Cardiovascular diseases (CVDs) are the leading cause of mortality and morbidity worldwide and the development of novel therapeutic treatments still remains a major research goal. The contribution of risk factors, such as cigarette smoking, diabetes, hypertension, and hyperlipidaemia, are well recognized as important players for the initiation of cardiac diseases, for which atherosclerosis is commonly shared by all CVDs [1]. Many cardiac disorders, such as heart failure (HF), ischemic/reperfusion (I/R) damage, and myocardial infarction (MI), are characterized by massive cardiac myocytes death [2]. On the other hand, adult human heart has limited capacity to replenish the loss of cardiomyocytes, having an extremely low regenerative ability after cardiac injury, despite some studies having suggested that human heart owns a certain degree of regenerative capacity [3]. Therefore, the loss of cardiomyocytes irreversibly damages the heart with a progressive decrease of its functionality and eventually develops into heart failure. In the context of such a scenario, the identification of the molecular mechanisms underlying cardiac diseases becomes mandatory and the possibility to capture the early events of disease development is crucial for the unraveling of mechanisms and/or markers that can act as potential targets against which to develop new therapeutic strategies. For decades animal models, especially rodents, have represented the model of election for studying human biology, development, and disease, based on the genetic and physiological similarities between the two species. Nonetheless, their phylogenetic relatedness has developed differently for humans and mice, making the experimental results obtained from animal models strikingly different from what is instead true for humans. Thus, although animal models have offered important contributions in understanding human biology and disease, they do not fully mirror the complexity of diseases as they are present in human systems and fail in the intent to translate the results of mice research to humans [4]. Moreover, many promising chemical compounds and drugs that perform well in preclinical animal studies fail in humans due to lack of safety and/or efficacy. Therefore, the use of human tissues would be the most reliable way for a precise understanding of the molecular underpinnings of human biology and pathology, resulting in more accurate and targeted therapies for many human disorders. The use of primary cells from living affected individuals is limited by the amount of tissue and its lifespan in culture, as well as the technical feasibility of accessing particular cell types such as neurons and cardiomyocytes. Human pluripotent stem cells (hPSCs), including embryonic stem cells (ESCs) and induced pluripotent stem cells (iPSCs), have the capability to self-renew indefinitely and to differentiate into derivatives of the three germ layers (ectoderm, mesoderm, and endoderm). These features make them very promising in a variety of basic research and clinical applications such as developmental biology [5], drug screening, disease modelling, and regenerative medicine. Unlike non-human animal models, tissue specific-derived PSCs offer an unprecedented platform for a comprehensive understanding of the molecular basis of CVDs [6]. The revolutionary discovery that somatic cells can be reprogrammed, via overexpression of a set of specific transcription factors, to induced pluripotent stem cells (iPSCs) [7,8,9], paved the way for the generation of patient-specific iPSCs [8,10]. iPSCs derived from healthy individuals or diseased patients carry the genome of their cell of origin and can be differentiated into any cell type, including cells not otherwise accessible, representing a powerful cell-based model system for human diseases, genetic investigations, drug screening, and personalized therapy [11]. Their human origin, pluripotency and ultimately their capability to differentiate into any disease-relevant cell type, and their epigenetic and genetic matching with the patient they are derived from are all features that make iPSCs the most reliable candidate for studying human disorders at cellular level. Moreover, genome-editing approaches can be used to repair and thus to rescue the disease phenotype in patient-derived iPSCs or to introduce pathologically relevant mutations in wild-type lines. To date, a wide number of different monogenic and complex human cardiac disorders have been modeled in vitro using iPSC technology providing new insights into disease mechanisms. The aim of this work is to address the most relevant scientific advances with respect to the use of iPSCs for cardiac disease modeling and to summarize the revolutionary potential of iPSC-derived cardiomyocytes (iPSC-CMs) for cardiac regenerative medicine.

## 2. Human Pluripotent Stem Cells: Applications, Power, and Limitations

**Figure 1:**
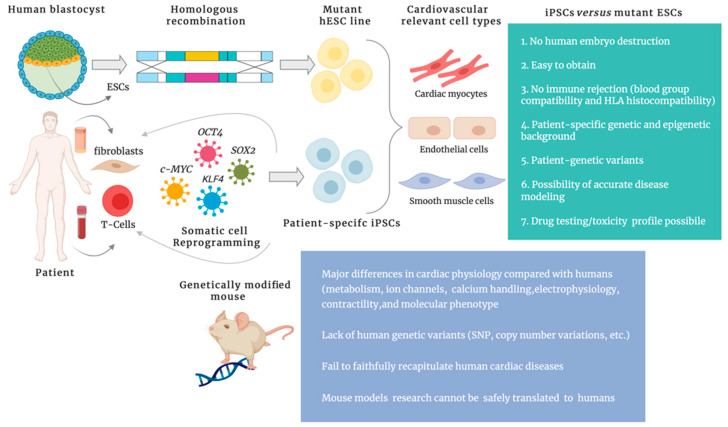
Advantages of induced pluripotent stem cells (iPSCs) over mutant embryonic stem cells(ESCs) and genetically modified mouse models.

Human pluripotent stem cells, including ESCs and iPSCs, have the ability to differentiate into derivatives of all three primary germ layers (ectoderm, mesoderm, and endoderm) [12], under specific culture conditions, and to self-renew indefinitely through mitotic cell divisions [13]. Based on these properties, these cells represent an inexhaustible cell source, offering an unprecedented and reliable model system for studying the molecular and genetic basis of human cardiovascular diseases, since they can be coaxed into differentiating into various relevant cell types, such as cardiomyocytes, smooth muscle cells, and vascular endothelial cells. ESCs are pluripotent stem cells derived from the undifferentiated inner cell mass (ICM) of a human embryo. Following their first isolation reported in 1998 [12], ESCs were predicted to provide a limitless cell source to interrogate human diseases as well as for supplying somatic cells destroyed by diseases and restoring normal function of damaged tissue. However, although very attractive, ESCs have some important limitations: only common, monogenic conditions can be captured using disease-specific ESCs derived from diseased embryos diagnosed via preimplantation screening [14]; ESCs are not genetically matched to the patients, thus, they need to undergo methods involving genome manipulation to generate a disease-specific model system. Moreover, their adoption in biomedical research has been dramatically slowed down by the ethical concerns surrounding the ESC derivation process that necessarily implies the destruction of the human embryo. The limited access to human embryos and the risk of immune rejection after transplantation into patients have hampered the promise of ESCs for biomedical research and cell therapy. Despite these limitations and the strict ethical control applied for their derivation, ESCs still remain the gold standard of pluripotency. Additionally, these cells have provided a robust cellular platform for the scientific community and, most importantly, have opened up the door to the development of iPSCs. iPSCs, the “artificial” counterpart of ESCs, are generated from adult somatic cells, such as skin fibroblasts, skin keratinocytes, peripheral blood cells, neuronal progenitor cells, or urine epithelial cells, through a process known as “cellular reprogramming”, consisting of forced expression of specific transcriptional factors including *OCT3/4*, *SOX2*, *KLF4*, and *c-MYC* [8], or some other combination of transcription factors such as *NANOG* and *LIN28* [9]. iPSCs and ESCs are similar in many aspects such as morphology, proliferation rate, gene expression profile, surface antigens expression, teratoma formation, epigenetic state of pluripotency, and telomerase activity [8]. As ESCs, iPSCs have the ability to proliferate indefinitely while maintaining their differentiation potential. Nonetheless, despite the high similarity between iPSCs and ESCs, there is still the matter of controversies concerning the extent of their real molecular and functional equivalence [15]. Comparative gene expression analyses of human iPSCs and ESCs have indeed revealed the presence of a small number of genes that are differentially expressed between these human pluripotent stem cell lines, suggesting that iPSCs might display significant differences of molecular profiles including genomic instability and epigenetic, non-coding and coding-RNA expression [16]. Additionally, a combined “multi-omics” study encompassing transcriptomic, proteomic, and phosphoproteomic profile analysis of reprogrammed iPSCs versus ESCs has demonstrated the presence of a small, but statistically significant, group of signaling pathways exclusively enriched in iPSCs, providing again evidence that reprogrammed cells may have a unique molecular signature, highlighting the complexity of human pluripotency [17]. Although concerns about the real equivalence between iPSCs and ESCs still exist, the effort of the scientific community to make these differences irrelevant as much as possible is undeniable. As ESCs, iPSCs also have some important limitations: genomic instability, interline variability, chromosomal variations [18], genetic mutations arising during the reprogramming process, and epigenetic memory reflecting the state of the somatic cell of origin which seems to be lost upon prolonged cell passages suggesting a subtle relevance of this memory [19]. Nonetheless, iPSCs hold remarkable advantages over mutated ESCs: (1) the strategy of somatic cell reprogramming provides an unlimited, easily accessible, pluripotent cell source; (2) the in vitro derivation of iPSCs eliminates the ethical issue linked to the destruction of human embryos; (3) iPSCs retain the same genetic background of the individual they are derived from and this is fundamental to avoid immune response [20]; (4) the human origin of iPSCs makes them a reliable cell source for transplantation. The advantages of iPSCs over mutant ESC lines and genetically modified mice are shown in Figure 1.

**Figure 2:**
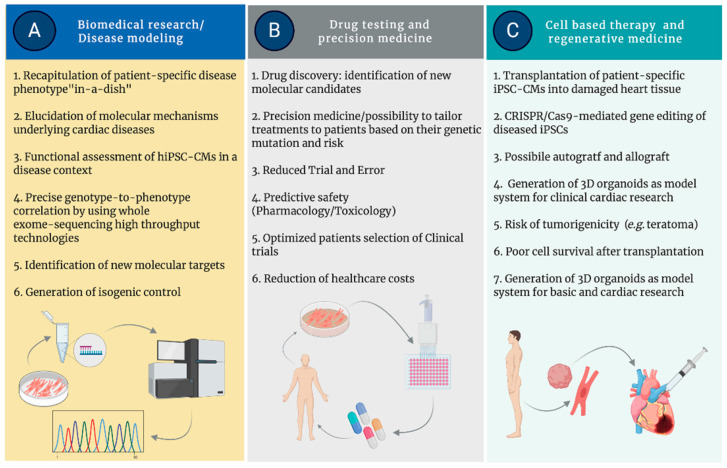
Major applications of iPSCs in research and medicine.

The wide range of clinical applications of iPSC technology is astounding: it allows the isolation of patient-derived cells carrying the genetic variants causing a given disorder, providing a human model for studying the disease “in-a-dish”. Moreover, the possibility of using iPSCs in regenerative medicine is the most ambitious goal for treating degenerative and progressive human diseases and developing patient-customized therapies and strategies for precision medicine. A summary of the most relevant applications of iPSC technology in cardiac research and medicine is shown in Figure 2.

## 3. iPSCs and Genome Editing

**Figure 3:**
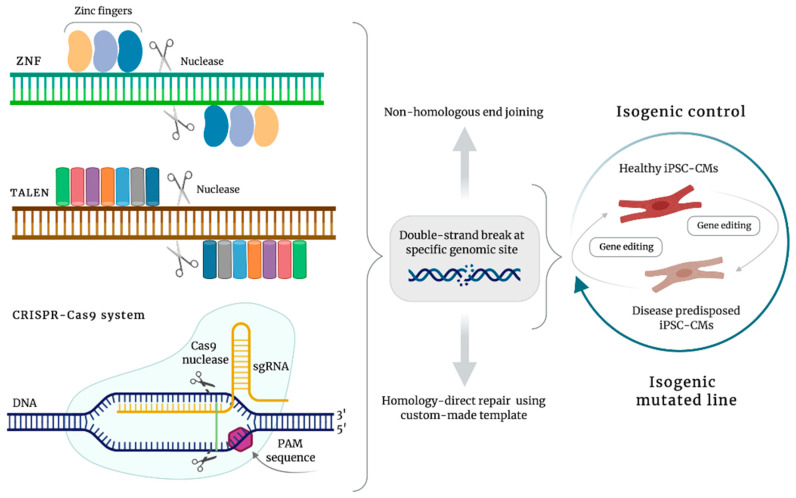
Application of genome-editing tools in human iPSC technology.

The combination of reprogramming technology to generate iPSCs and the development of methods to efficiently differentiate iPSCs into many cell types has revolutionized the way human diseases are modeled, allowing a growing knowledge of the pathophysiological mechanisms underlying cardiac diseases. Ever since, the rapid accumulation of information about the mechanisms responsible for cardiac diseases has quickly required strategies to modify the genetic background of patient-specific iPSC-derived cells, to generate healthy cells or to introduce diseases-specific variants in wild-type iPSCs. Advances in genome editing techniques have led to the generation of isogenic controls and mutated cell lines particularly relevant in the case of pathologies in which a single mutation is responsible for the disease phenotype. In a context in which one of the major applications of iPSC technology is in vitro disease modeling, the development of efficient genome-editing strategies represents a major goal. These technologies are extremely fascinating and useful when modeling very rare diseases for which the accessibility to patients with the disease of interest is nearly impossible or in the case in which the number of available patients is too low to make the study reliable. These are situations in which the introduction of disease-causing mutations in wild-type iPSCs using genome-editing approaches significantly contributes to a comprehensive understanding of the pathophysiology of rare diseases, allowing the creation of human model systems using healthy iPSC lines available in a biobank. The power of iPSC technology for investigating complex diseases is definitely advantageous and unprecedented, but a good disease model needs a good control. Very often, iPSCs generated from healthy relatives or healthy genetically unrelated subjects have been used, inadequately, as standard control. The phenotypical differences seen in diseased iPSC-derived cells and derivatives from control iPSCs may be the results of a different genetic background rather than disease-specific variants. The coupling of iPSC technology and genome editing has overcome these limitations. The main tools allowing precise genomic manipulation in wild-type and diseased iPSCs are represented by zinc-finger nucleases (ZNFs) [21], transcription-activator like effector nucleases (TALENs) [22], and clustered regularly interspaced short palindromic repeats (CRISPR)/CRISPR-associated protein 9 (Cas9) system [23] (Figure 3). All these techniques aim to introduce double-strand DNA breaks (DSBs) at a precise and desired site in the genome. DSBs are repaired by means of two main mechanisms: nonhomologous end joining (NHEJ) and homologous recombination (HR) (Figure 3).

NHEJ is a highly efficient, error prone process that can introduce insertions or deletions. HR is a more reliable but less efficient method, based on the use of a homologous template (either sister chromatid or chromosome or a synthetic template), allowing the precise introduction of a specific change into the human genome. The change can be targeted either to introduce a specific disease-causing mutation in healthy iPSCs or to correct a preexisting genomic variant to generate an isogenic control line. Among the genome-editing technologies, CRISPR-Cas9 is nowadays the tool of choice to generate genetically modified iPSCs, based on its low cost, simple construction, and high fidelity. The possibility to couple the versatility of iPSCs together with CRISPR-Cas9 technology in a single impactful experiment is proved to have redrawn our approach to stem cells biology and biomedical research, allowing the establishment of a powerful approach to derive isogenic lines, meaning that diseased iPSCs and the control lines are genetically matched and they only differ in the disease-causing variant. In the past few years, the application of CRISPR-Cas9 technology in disease modeling has allowed the generation of isogenic iPSC-based disease models for several cardiomyopathies and channelopathies, including dilated cardiomyopathy (DCM) [24], Barth syndrome (BTHS) [25], long QT syndrome (LQTS) [26], Brugada syndrome (BS) [27], and left ventricular non-compaction (LVNC) [28]. However, as happens for the majority of technologies involving human cells and genome manipulation, CRISPR-Cas9 technology has also raised fundamental ethical concerns. If on one side CRISPR-Cas9 has permitted a variety of genomes to become available, on the other side, the technology has pointed out several issues, with particular regards to the extent of its applications on all human genomes as well as germ cell line modifications, and other concerns elegantly reviewed elsewhere [29,30].

## 4. Differentiation of Cardiac Cells from iPSCs

For iPSCs to dominate a wide spectrum of biomedical fields, their effective differentiation into specific cell types is of extreme importance. In vitro CM differentiation from iPSCs is achieved by modulation of signaling pathways known to be involved in cardiac development during normal embryogenesis [31]. To date, there have been three main strategies developed to obtain functional CMs from iPSCs: (1) co-culture with visceral endoderm-like cells (END-2); (2) embryoid body (EB)-based differentiation, and (3) two-dimensional culture. During embryonic development in vivo, visceral END-2 releases factors that lead to cardiac differentiation of the nearby mesoderm [32]; this discovery was the basis of the co-culture strategy in which PSCs cultured either in the presence of END-2 cells or in END-2-conditioned medium enter cardiac fate. Although this protocol was successfully applied to both ESCs [33] and iPSCs [34], the CM yield is very low (less than 10%) [35]. EB-based differentiation is a serum-mediated three-dimensional method relying on the capability of PSCs to form floating cell aggregates when cultivated as single cells in low attachment substrate. These aggregates, known as embryoid bodies (EBs), spontaneously produce derivative cells of all the three germ layers [36]. However, the EB-differentiation procedure, due to the presence of serum, suffers from low reproducibility and inter-line variability [37]. Serum was later replaced by cytokines and growth factors known to be involved in heart development such as Wnt proteins [38], bone morphogenetic proteins (BMPs) and activin A [31,39], and Notch signals [40], together with their corresponding inhibitors [41,42]. Different small molecules have been tested for their ability to promote in vitro cardiac differentiation; activators (CHIR99021) and inhibitors (IWR, XAV, IWP2) of the Wnt pathway have been proved to increase cardiac differentiation [43]. However, this strategy requires a high number of starting cells and has a low efficiency. To overcome the limitations of methods based on EB formation, differentiation protocols have been developed based on cell monolayers but with the use of the same molecular factors described for EB differentiation [44,45]. A monolayer-based strategy allowed significant improvement of the yield of cardiac differentiation and the phenotype of derived cardiac cells exhibiting typical features of ventricular, atrial, or nodal cardiomyocytes [46]. The “matrix sandwich” method is a modification of monolayer assay, consisting of covering confluent iPSCs, previously treated with specific growth factors and cytokines to induce cardiac differentiation, with an overlay composed of matrix (i.e., Matrigel) and culture medium. This method relies on the pivotal role that the extracellular matrix plays in the differentiation process leading to high CM purity and yield [47]. Other differentiation methods require two steps: during the first step iPSCs are induced to differentiate into cardiac progenitor cells (CPCs), which in turn can be further differentiated into different cellular fates including CMs, smooth muscle cells (SMCs), and endothelial cells [48,49]. Moreover, it was recently demonstrated that induced CPCs can be directly generated using mouse fibroblasts, skipping the intermediate stage of iPSCs [50,51]. Although there are currently available protocols providing a differentiation efficiency of up to 80% or more in terms of CM purity [46,49,52], all the so far reported strategies show major limitations such as heterogeneity and immaturity of the cardiac population [53]. The low purity and high heterogeneity of the differentiated CM population constitutes an important obstacle for their use in cell-based therapy that requires efficient purification methods to enrich the cardiac population. So far, several studies have developed efficient isolation methods based on the identification of specific cardiac markers such as SIRPA and VCAM1 [54,55,56]. Other studies have instead developed protocols to differentiate human iPSCs into specific subtypes of functional cardiac cells, such as atrial-, ventricular- [57,58,59], nodal-like [60] and pacemaker cells [61]. Although the cardiac cells obtained from iPSCs can start beating very early during differentiation, they resemble, morphologically and functionally, fetal cardiomyocytes. iPSC-CMs display a disorganized morphology, reduced contractile capacity, alteration of glycolytic metabolism, abnormal electrophysiological properties, and reduced automaticity [62]. This immaturity renders adult-onset heart disease modeling very challenging, owing to the uncertainty regarding the ability of relatively immature iPSC-CMs to fully recapitulate adult disease phenotypes or as a function of aging, while the understanding of early-stage pathological events is not affected by low iPSC-CM maturity. Moreover, the incomplete maturity of iPSC-derived CMs could narrow the effectiveness of these cells in mimicking the pathology, e.g., if this is caused by a gene mutated postnatally, with negative impact on their usefulness for studies on drug effect/toxicity. In order to improve the differentiation strategy of iPSCs toward the generation of a high mature and homogeneous cardiomyocyte population, new differentiation methodologies and technical modifications have been proposed. A long culture period (80–120 days) results in multinucleated iPSC-CMs exhibiting mature sarcomeres and increased electrophysiological properties compared to 20–40-day-old CMs [63]. This higher grade of maturity of long-term culture CMs is strictly related to mitochondrial metabolism regulation, which is necessary for energy production and increased cell contractility [64]. Other methods aimed to improve CM maturation include addition of T3 hormone [65] or dexamethasone [66] in culture medium and stressing CMs with mechanical and electrical stimuli [67]. Among the methods developed to increase the maturation of iPSC-CMs, in vivo environments provided the most mature phenotype of iPSC-CMs [68,69]. Currently, nanotechnology-based approaches offer new perspectives in many fields of biomedical research, including cardiovascular research [70]. Three-dimensional scaffolds produced starting from natural or synthetic materials and functionalized to reach specific mechanical and chemical features can be used for direct iPSC differentiation into cardiomyocytes. Scaffolds designed for cardiac differentiation should possess specific properties such as good elasticity to allow cardiac cell contraction and properties to allow cardiomyocytes to arrange in a polarized and organized structure typical of native myocardium. These properties are retained for example by poly (vinyl alcohol) [71], polyethylene oxide [72], poly(lactic-co-glycolic acid) [73], and poly(caprolactone) [74]. CMs cultured on 3D structures show an enhanced calcium signaling respect to monolayer culture [75]; however, it is mandatory to combine this method with electrical and physiological stimulation to obtain cardiac cells with a complete degree of maturity [76]. The number of differentiated cells obtained using classical culture methods represents an additional shortcoming for the application of iPSCs in cell therapy, given that up to one billion CMs need to be transplanted within the infarcted myocardium to replace damaged tissue [77]. Large-scale production of CMs from iPSCs can be achieved using bioreactors that make the process scalable and reproducible via the continuous control and stabilization of culture parameters [78]. Bioreactors create a dynamic suspension culture in which there is a constant flow of nutrients and homeostasis of pH and oxygen levels [79]. iPSCs cultured in a spinner flask form aggregates that can be used as starting materials for CM production after treatments with molecules acting on the Wnt pathway. The spinner flask methods allow critical variables to be tightly supervised, such as aggregate size and cytokine release, augmenting differentiation efficiency [80]. The culture in the suspension of cells that are adhesion-dependent for survival and proliferation can be obtained through the use of supporting matrices known as microcarriers [81]. Laco et al. (2020) developed a microcarrier culture system in a tank bioreactor that allowed scalable iPSC expansion and CM differentiation and purification, reaching a yield of ~40 CMs per iPSC seeded after 22 days in culture [82]. A future intension of bioreactor use will be the production of large amounts of high quality CMs in GMP manufacturing, to improve their use in clinical practice.

## 5. Drug Discovery and Personalized Medicine

Remarkable progress in the development of new cancer therapies has dramatically changed the landscape of treatment approaches for several malignancies, thus contributing to increased patient survival. Cardiovascular toxic effects of cancer therapeutics and radiation therapy are the epitome of concerns for cancer treatments. Furthermore, the cardiotoxicity spectrum has broadened to include myocarditis with immune checkpoint inhibitors and cardiac dysfunction in the setting of cytokine release syndrome with chimeric antigen receptor T cell (CAR-T) therapy. An increase in the incidence of arrhythmias related to inflammation such as atrial fibrillation can also be expected, in addition to the broadening set of cancer treatments that can induce prolongation of the QT interval. Therefore, cardiologists have to be familiar not only with cardiotoxicity associated with traditional cancer therapies, such as anthracycline, trastuzumab, or radiation therapy, but even more with an increasing repertoire of additional therapeutics. The latest developments at the juncture of cardiology, oncology, and hematology have been recently widely discussed [83]. iPSC technology integrated with genome-wide association studies (GWAS), next generation DNA and RNA sequencing (NGS), and chemical libraries, represents a powerful platform for cell-based screening and validation of novel therapeutic compounds and/or new targets predicted by in silico analyses [84], or for the screening of existing drugs that could be repurposed for new medical uses. For example, thanks to iPSC-based screening, it was possible to identify a new application for ezogabine, an anti-epileptic drug, in amyotrophic lateral sclerosis (ALS) treatment [85] and to highlight the beneficial effects of *MAP4K4* gene silencing on an iPSCs-CMs model of ischemic injury [86]. In addition, several high throughput techniques are employed for the assessment of the drug effects on cardiac physiology. Action potential is recorded using automated planar patch clamp platforms [87] or microelectrode arrays [88]; Ca^2+^ concentration is quantified using Ca^2+^-sensitive fluorophores [89]; cardiomyocyte contractility can be measured using muscular thin film [90] or dynamic monolayer force microscopy [91]. Preclinical drug screenings on iPSC-derived CMs offers the opportunity to promptly discover the cardiovascular toxic effect of specific drugs [92]. For instance, iPSC-CMs derived from patients with breast cancer have been used as a model to study the cardiotoxicity induced by doxorubicin. CMs treated with doxorubicin showed decreased cell viability and alterations in mitochondrial function and calcium handling, demonstrating that patients with breast cancer are more prone to be affected by doxorubicin-induced cardiotoxicity [93]. Cardiotoxicity due to repeated exposure to doxorubicin has been documented also in iPSC-CMs derived from healthy subjects [94]. A similar approach was applied to investigate the cardiotoxicity of twenty-one tyrosine kinase inhibitors (TKIs) used in cancer therapy. The in vitro high-throughput screening performed on iPSCs-CMs derived from healthy individuals and oncologic patients allowed the identification of two TKIs against the vascular endothelial growth factor (VEGF) and platelet-derived growth factor (PDGF) receptors as a mechanism for induced cardiotoxicity [95]. Another chemotherapeutic agent known for its cardiotoxicity is trastuzumab [96]; by using iPSC-CMs, researchers identified mitochondrial and energy metabolism dysfunctions as mechanisms responsible for trastuzumab-driven impairment of myocardium contraction [97]. Notably, since iPSCs retain the patient’s genetic background, thus mirroring the individual’s response to the drug, they provide a robust platform for the development of precision therapies in the so-called “personalized medicine”. An example of personalized treatment of cardiac channelopathies is represented by the study of Mehta et al. (2018) in which iPSC-CMs were obtained from long-QT syndrome type 2 (LQT2) patients carrying Class 1 and Class 2 mutations in the *KCNH2* gene. The treatment of CMs with the channel modulator lumacaftor led to a recovery of the pathological phenotype in CMs with Class 2 mutations but not in those affected by Class 1 mutations, showing the importance of genetic background in the pharmacological response [98]. Even if the use of patient-specific iPSC-based platforms in drug discovery and personalized medicine is advantageous for many aspects, they are not yet completely affirmed in the pharmaceutical industry due to the time-consuming nature and high cost of iPSC production and differentiation processes [99]. To overcome this issue, Solomon and colleagues have proposed the establishment of a biobank containing several iPSC lines reprogrammed from healthy donors carrying different combinations of human leukocyte antigen (HLA) alleles in order to provide a shared and economic starting point from which iPSC-derived differentiated cells are made available to HLA-matching individuals for drug investigations and precision medicine [100].

## 6. Cardiac Regenerative Medicine

Despite the remarkable progress in therapeutic developments, CVDs still tremendously impact global health. The current available pharmacological approaches for HF treatment have increased the lifespan but are inefficient to repair the cardiac tissue irreversibly damaged by cardiomyocyte loss. HF is the end-stage of cardiovascular disease progression for which, other than heart transplantation, there are no therapies addressing the loss of cardiomyocytes. However, heart transplantation is not always applicable because of the limited number of compatible donors. Additionally, the procedure is very risky with very important consequences (rejection of the donor heart, primary graft failure, infection, and even death). The discovery of patient-specific iPSCs together with their potential to be differentiated into cardiomyocytes has profoundly changed cardiac clinical practice and has produced a revolutionary alternative to heart transplantation. iPSC-derived CMs are thus considered a promising and extremely attractive cell source for individuals suffering from severe heart failure. iPSC-based therapy has the potential to restore cardiac function via substitution of damaged or dead cardiomyocytes. The regenerative capability of the human heart is still a controversial question in the scientific community: for a long time it has been thought that the regeneration capability of the human cardiac tissue decreases immediately after birth and postnatal cardiac cells have been considered “terminally differentiated” cells, restraining in the G0 phase of the cell cycle during lifespan [101]. In 2009, the presence of cardiomyocyte renewal, although at low rate, in the mammalian heart was reported [3]. Among the different stem cells available for potential application in cardiac regenerative medicine there are skeletal myoblasts [102], bone-marrow derived cells [103], endothelial stem cells [104], mesenchymal stem cells [105], adipose-derived stem cells [106], cardiac stem cells [107], and pluripotent stem cells [108,109]. iPSCs, with their ability for self-renewal and their capability to differentiate into any cell type, without the risk of post-transplantation immune rejection, represent the ideal candidate for regenerative medicine [110]. Different studies have demonstrated the benefit and efficacy of iPSC-derived CMs in restoring compromised cardiac function and morphology [111,112,113,114]. However, before iPSCs can be safely used in clinics, some limitations need to be addressed and solved. One of the main risks with in vitro-generated cardiomyocytes is their tendency to form teratomas after transplantation due to the presence of undifferentiated cells. Therefore, the purification of the iPSC-CM population before their transplantation is critical [115]. An established method for the purification of iPSCs-CMs from undifferentiated cells is based on the utilization of glucose-depleted and lactose-supplemented culture medium, because PSCs do not survive without glucose [116]. Another important requirement toward regenerative medicine is the mature phenotype of iPSC-CMs. In order to be used in an injured heart, iPSC-CMs need to acquire an adult phenotype, especially in terms of electrophysiology and calcium handling. To date, several methods aimed at enhancing the maturation of iPSC-derived CMs (electrical stimulation, 3D cardiac tissue remodeling or co-culture with non-CMs cells to re-create the cardiac microenvironment, long-term culture in vitro, mechanical stimulation, and others) are under investigation [117]. However, many of these strategies are limited by the lack of a large-scale applicability. The criteria to which iPSC technology must respond before being applicable in cell transplantation therapy are: (1) overcoming the heterogeneity of the iPSC-CM population, which is composed of atrial-, ventricular-, and nodal-like CMs, and this might be problematic as there is evidence that the transplantation of nodal cells to a damaged heart could induce arrhythmias; (2) the cells to be transplanted need to be highly numerous in order to obtain a good engraftment rate; (3) the method used for the introduction of iPSCs-CMs into the damaged heart needs to be accurately chosen; in fact, previous data have shown that CMs directly injected into the injured heart as a single cell suspension have a very low survival rate, while the transplantation of CMs as cell aggregates (cardiac spheroids) or in form of cardiac cell sheets is more efficient [116].

Several studies on animal models have documented the encouraging and enormous potential of PSCs in regenerative medicine. Shiba et al., for instance, have transplanted human ESC-CM into the myocardium of a guinea pig model of cardiac injury, obtaining an improvement of mechanical and electrical function together with a decrease of ventricular arrhythmias [112]. In 2007, Caspi and collaborators used rats as an animal model for ESC-CM-based therapy for injured myocardial repair. The implantation into the rat’s myocardium of beating CMs derived from ESCs isolated from contracting embryoid bodies (EBs) resulted in a stable cardiomyocyte graft and in the attenuation of the myocardium deterioration [118]. Another study demonstrated an improvement of cardiac function after transplantation of human ESC-derived cardiomyocytes into a rat’s infarcted myocardium [39]. Moreover, the work of van Laake et al., used a mouse model of infarcted heart to show that the transplantation of ESC-CMs into a damaged cardiac area resulted in an improvement of heart function after four weeks [119]. Recently, this technology has also been applied on large animals such as pigs, as shown in the work of Kawamura et al., who reported an improvement of the cardiac function in a pig myocardial infarction model after transplantation of human iPSC-CM sheets [120].

The potential of iPSC technology in cardiac regeneration is currently under evaluation in Japan; since 2018, the clinical trial, driven by Yoshihi Sawa, has used thin sheets of cardiac tissue derived from iPSCs in diseased human hearts to help cardiac regeneration [121].

## 7. iPSCs in Cardiac Disease Modeling

In vitro disease modeling is one of the most speculated about fields using iPSC technology. Modeling human cardiac disorders enables definition of the functional and molecular mechanisms underlying a disease and creates the possibility to develop new therapies. The first lines of iPSCs from patients harboring monogenic and complex genetic diseases were established in 2008; one year later, iPSCs were generated from a human specimen [10]. This pioneering publication has rapidly been followed by a growing body of scientific literature. iPSC-based disease modeling has dramatically influenced cardiovascular medicine, offering the opportunity to understand the pathological mechanisms of cardiac diseases and to develop novel effective therapies. This has greatly attracted the scientific community, providing an unprecedented opportunity to recapitulate human monogenic and complex cardiac diseases in vitro. Before the advent of iPSC technology, the severity of CVDs together with the lack of efficient treatments rather than transplantation pushed researchers to develop model systems of cardiac diseases comprising animal models for in vivo studies, in vitro cellular models based on the use of stem cells, primary cells, and various cell lines, and computational studies [122]. The potential of iPSCs and their capability to differentiate into cardiac relevant cell types, including cardiomyocytes, smooth muscle cells, and vascular endothelial cells, as well as and their genetic match to the patient they are derived from, offers a large spectrum of possibilities for the establishment of a robust in vitro model of the disease. iPSC-derived CMs share the same genetic and molecular blueprints as primary human CMs, along with mechanical and electrophysiological properties. To date, a wide range of cardiac diseases including long QT syndrome [123], Leopard syndrome [124], Brugada syndrome [125], catecholaminergic polymorphic ventricular tachycardia [126], arrhythmogenic right ventricular cardiomyopathy/dysplasia [127], dilated cardiomyopathy [128], left ventricular non-compaction [28], hypertrophic cardiomyopathy [129], Andersen-Tawil syndrome [130], and Timothy syndrome [131] have been modeled using iPSC technology. Cardiac diseases are traditionally divided into three main groups: channelopathies, structural cardiomyopathies, and others disorders that do not fit in as channelopathies or structural cardiomyopathies. Among cardiac diseases that are not recognized as channelopathies and/or structural cardiomyopathies, there are several metabolic disorders with cardiac phenotypes. Some of them, such as Friedreich’s ataxia [132], Barth syndrome [25,133], fatty acid oxidation disorders, and Pompe diseases [134] have successfully been translated to iPSC-CM-based models. Ion channelopathies are perhaps the form of cardiac disease with the most well-established iPSC-based disease models.

### 7.1. Long QT Syndrome

Long QT syndrome (LQTS) is the most common ion channelopathy, typically characterized by a prolongation of the QT interval (repolarization phase) on the EGC, increased risk of ventricular arrhythmias (i.e., *torsades de pointes*), and sudden cardiac death [135]. So far, there are at least ten different subtypes of LQTS according to the underlying channel or gene mutation, and research based on iPSC-derived CMs has mainly focused on modelling LQST1, LQTS2, and LQST3, although others, such as LQTS7 (Andersen-Tawil syndrome), TS, LQTS8 (Timothy syndrome), and LQTS14/15 (calmodulinopathies), have also been investigated with iPSC technology. LQTS develops as a genetic disorder with an autosomal dominant inheritance trait, and mutations in *KCNQ1*, *KCNH2*, and *SCN5A* genes are causative of LQTS [136]. The first LQTS type 1 (LQT1) disease model based on iPSC-CMs was reported by Moretti et al., in 2010. This elegant work highlighted the potential of iPSC technology to model cardiac diseases and to test novel therapeutic strategies [137]. The authors generated iPSCs from two members of a family carrying an autosomal missense mutation (R190Q) in the *KCNQ1* gene. The *KCNQ1* gene encodes for a subunit of the ion channel responsible for conducting the adrenergic-sensitive, slow outward potassium current, I_Ks_. The atrial and ventricular-type hiPSC-derived CMs had a significant reduction (70–80%) in I_Ks_ current and impaired activation-deactivation of the channel compared to the healthy counterpart. Moreover, it was also demonstrated that beta-blockade treatment of LQT1 iPSC-CMs had a protective role against catecholamine-induced tachyarrhythmia. Afterward, these data were confirmed via analysis of iPSC-CMs carrying a different *KCNQ1* mutation [138,139].

Electrophysiological measurements on iPSC-CMs generated from patients affected by a LQTS1 carrying mutation in *KCNQ1* have shown a clear prolongation of the action potential (AP) into atrial-like and ventricular-like cells and a reduction of I_Ks_ current due to a decreased number of functional KCNQ1 channels in the sarcolemma [137,138,139]. Moreover, an association mechanism was identified between mutated *KCNQ1* genes and abnormalities in Ca^2+^ handling, and treatment with Ca^2+^ antagonists restored the electrophysiological phenotype [140].

Loss-of-function mutations in the *KCNH2* gene (known also as hERG), a K^+^ channel involved in the rapid delayed rectifier current I_Kr_, are common in the LQTS2 phenotype [141]. iPSC-CMs derived from patients with a mutation in the *KCNH2* gene showed an increase in action potential duration (APD) and important arrhythmogenic events due to a reduction of the cardiac potassium current I_Kr_ [142]. The treatment of CMs with I_Kr_ blocker E-4031 worsened the pathological phenotype, while administration of nifedipine (a Ca^2+^-channel blocker) or pinacidil (a K_ATP_-channel opener) resulted in shorter AP [143]. LQT2 iPSC-CMs treated with isoprenaline also showed early after depolarization (EADs) events in respect to control CMs, and drugs that enhance potassium channel activity can restore AP normal properties. In LQTS2 iPSC-CM models, an increase of the action potential duration (APD) has been observed [144]. The phenotype has been recapitulated in vitro: an alteration of KCNH2 function, due to the presence of gene mutations, led to a decrease of the I_Kr_ current and the development of arrythmias [145]. In 2013, Bellin et al., reported a mutation (N996I) in KCNH2 responsible for a moderate growth of the APD without early-after depolarization (EAD) [142]. LQTS2 hiPC-CMs allowed the discovery that the reduction of I_Kr_ is caused by protein-displayed trafficking abnormalities due to alterations in the glycosylation of KCNH2. Treatment of CMs with calpain and proteasome inhibitors was able to restore the membrane localization of mature KCNH2 channels and to rescue the electrophysiological features of the cardiac cells [146]. Even for an LQTS2 model, the assumption that Ca^2+^ antagonists could counteract Ca^2+^ handling alterations was confirmed [147]. Gain-of-function mutations in the *SCN5A* gene result in LQTS3 [148]. This gene encodes for the cardiac Na^+^ voltage gated sodium channel Na_V_V1.5, which is the most important ion channel receptor localized at the heart level, designated for the control of Na^+^ currents determining the fast upstroke of cardiac action potential [149]. In this syndrome, there is the risk of *torsade de pointes*, caused by a delay in the repolarization of myocardium after a heartbeat [150]. LQTS3 iPSC-CMs recapitulated the disease phenotype showing an increase of persistent Na^+^ current during the plateau and repolarization phase of the AP current [151,152], and sodium channel blockers positively acted on CM arrhythmia [153,154]. LQTS3 in iPSC-CMs is characterized by the progressive inactivation of the sodium channel [151] or a rapid channel rescue from the inactivation [153]. Instead, loss-of-function mutations are responsible for the onset of Brugada syndrome (BrS) associated with upstroke velocity of the AP [155]. In particular, the generation of a BrS iPSC-CM model, following the isolation of fibroblasts derived from a patient with SCN5A-1795insD, showed a decrease of sodium channel function [156]. Andersen-Tawil syndrome (ATS), also called LQTS7, is an autosomal dominant potassium channelopathy characterized by gene mutations of *KCNJ2* (or ATS1). The syndrome is associated with periodic paralysis, ventricular arrythmias, elongation of the QT interval, and skeletal features [157]. *KCNJ2*, localized on human chromosome 17, encodes for the inward-rectifying potassium channel (K_ir_2.1) involved in the control of inward-rectifier potassium current (IK1) [158]. Mutations of this gene produce loss of function and dominant-negative suppression actions on the Kir2.1 protein with consequent alteration of the cardiac and skeletal muscle excitability [159]. In particular, three mutations of *KCNJ2* have been investigated through the application of iPSC-CMs in order to understand the pathological mechanism underlying ATS. ATS iPSC-CM models reflect the alterations of electrophysiological characteristics of the disease defined by arrhythmia and abnormal Ca^2+^ release that might be modified by the assumption of antiarrhythmic agents such as flecainide, a modulator of sodium-calcium exchanger (NCX) current [130]. Timothy syndrome (TS), or LQTS8, is a rare multisystem disorder characterized by prolongation of the QT interval, syndactyly, seizures, behavior abnormalities, immunodeficiency, and hypoglycemia [160]. TS is caused by a mutation in the *CACNA1C* gene encoding for the sarcolemma voltage-gated Ca^2+^ channel (Ca_V_1.2.). This protein is the most important L-type calcium channel localized at heart level which is involved in the generation of cardiac action potential and in excitation-contraction coupling [161]. TS has been modeled on iPSC-CMs and the detailed investigations performed have shown a slow intrinsic beating rate in ventricular iPSC-CMs, in line with the bradycardic events of TS patients. This phenotype is due to abnormalities in Ca^2+^ handling and can be recovered using roscovitine treatment [131].The possibility to reproduce in vitro the TS phenotype using iPSC-CMs, derived from patients affected by this disorder, has allowed the identification of new potential pharmacological substances useful for the treatment of the syndrome [131]. Calmodulinopathies, also indicated as LQTS14 and LQTS15, are caused by mutations of three calmodulin genes, CALM1, CALM2 and CALM3, that encode for CaM proteins. Alterations of these proteins produce defects in the Ca^2+^ binding protein activity of calmodulin leading, especially in children and young people, to severe arrhythmias for which there is no effective treatment [162]. iPSC-CMs were obtained from patients carrying a different mutation in the CALM gene; these models recapitulated the pathological features of calmodulinopathies, including slow inactivation of I_CaL_ current (responsible for the main entry of calcium in CMs) and massively prolonged APDs [163]. Silencing of the CALM gene in LQTS iPSC-CMs using CRISPR technologies resulted in the recovery of a healthy phenotype, showing a new kind of cure for calmodulinopathies. In 2016, Limpitikul el at. applied iPSC-CMs to study and understand calmodulinopathy in patients carrying the point mutation D130G in CALM2; it was noticed that the LQTS phenotype is linked to prolonged APD, slow I_Ca_ inactivation, and myocyte Ca^2+^ abnormalities [164].

### 7.2. Leopard Syndrome

LEOPARD syndrome (LS), also known as Noonan syndrome (NS), is a rare autosomal dominant complex dysmorphogenetic disorder mainly characterized by skin, facial, and cardiac abnormalities. LEOPARD is an acronym for the major features of this complex disorder and stands for lentigines, electrocardiographic conduction abnormalities, ocular hypertelorism, pulmonary stenosis, abnormal genitalia, retardation of growth, and sensorineural deafness. Genetically, LEOPARD syndrome associates, in more than 80% of patients, with mutations in the *PTPN1* gene that encodes the protein-tyrosine phosphatase non receptor 1 that catalytically inactivates the tyrosine phosphatase SHP2 (Src-homology 2 domain-containing phosphatase 2), which plays a critical role during development [165]. SHP2 regulates important intracellular signaling pathways such as Ras/MAPK, phosphoinositide-3 kinases (PI3K)/Akt, target of rapamycin (TOR) kinase, and JAK/STAT by specific tyrosine residue dephosphorylation. Carvajal-Vergara and collaborators have successfully generated iPSCs from LS patients carrying mutations in the *PTPN1* gene. Patient-specific iPSCs were differentiated into cardiomyocytes that were shown to be larger, with a highly structured and organized sarcomere and a preferential nuclear localization of NFATc4 (transcription factor associated with a calcineurin-mediated hypertrophic signaling, defined as nuclear factor of activated T cell 4), which is responsible for the hypertrophic phenotype seen in LS patient’s CMs compared to their healthy counterpart [124].

### 7.3. Catecholaminergic Polymorphic Ventricular Tachycardia

Catecholaminergic polymorphic ventricular tachycardia (CPVT) is a rare inherited cardiac disease predominantly caused by an autosomal dominant mutation occurring in the gene encoding the cardiac ryanodine receptor (RYR2). The disease is devastating with lethal consequences mediated by tachyarrhythmias occurring under exercise or emotional stress [166]. CVPT can be classified into two main subtypes: CPTV1 and CPTV2. Subtype 1 (CPVT1) is generated by gene mutations on the ryanodine receptor Type 2 gene (RYR2), while a mutated calsquestrin-2 (CASQ2) gene is responsible for subtype 2 (CPVT2) [167]. Both genes are necessary for the control of cardiomyocyte calcium handling: RYR2 is indispensable for the flow out of the sarcoplasmic reticulum (SR) in case of the depolarization process, instead, CASQ2 is a Ca^2+^-binding protein essential in the sarcoplasmic reticulum. Many different iPSC-based models of CPVT have been developed and all have succeeded in recapitulating the arrhythmogenic phenotype seen in patients. Jung and Moretti generated iPSCs from a patient diagnosed with familial CVPT. The cardiac cells derived from these patient-specific iPSCs displayed elevated diastolic Ca^2+^ concentrations, a reduced Ca^2^ content into the SR, and an increased susceptibility to arrhythmias, which are all disease-associated features occurring under catecholaminergic stress. Additionally, the authors described a protective role of dandrolene in CPVT cardiomyocytes [168]. Another group showed, using patch-clamp recordings of CPVT iPSC-CMs, delayed afterdepolarizations (DADs) both during spontaneous beating and in response to adrenaline, together with early afterdepolarizations coupled with aberrant Ca^2+^ cycling, which is typical of CPVT [169]. More recently, Sasaki and coworkers provided an iPSC model system of CPVT, recapitulating the disease phenotype and the antiarrhythmic effect of S107 as an approach for drug testing [170]. β-blockers are widely used to eliminate stress-induced ventricular arrhythmias occurring in patients suffering from CPVT, but this treatment is non-effective for some patients. In addition, iPSC-CMs from a CPVT patient respond positively to treatment with flecainide, demonstrating again the power of iPSC technology in capturing basic drug response effects [171].

### 7.4. Arrhythmogenic Right Ventricular Cardiomyopathy

Arrhythmogenic right ventricular cardiomyopathy (ARVC) is an inherited heart muscle disorder pathologically characterized by progressive fibrofatty replacement of the myocardium and loss of cardiomyocytes, predominantly in the right ventricle. These hallmarks associate with ventricular tachycardia, life-threating arrythmias, and sudden cardiac death in young people and athletes. A clinical picture of the ARVC disease was depicted by Thiene and collaborators [172]. ARVC has been defined as the “disease of the desmosome”, since genes encoding desmosomal proteins (plakoglobin, plakophilin, desmoglein, desmocollin, and desmoplakin) play a critical role in the disease pathogenesis. Nearly 50% of ARVC cases have a genetic origin and the mutated traits are inherited in an autosomal dominant way. In 2013, Kim and collaborators established an iPSC-based model by reprogramming skin fibroblasts from two ARVC patients harboring a mutation in the *PKP2* gene. In their study, the authors succeeded in establishing an in vitro model of ARVC by inducing an adult-like metabolism accompanied by the activation of peroxisome proliferator-activated receptor gamma (PPAR-γ), a master regulator of adipogenesis and lipogenesis in beating embryoid bodies (EBs) [173]. Caspi et al., applied iPSC technology to generate iPSC-CMs from two patients with plakophilin-2 mutations. CMs from both patients were reported to have an altered desmosomal structure that positively correlated with lipids accumulation, increased levels of PPAR-γ, and upregulation of Wnt and PPARγ signaling pathways. Moreover, authors showed that treatment of diseased iPSC-CMs with BIO (a GSK-3β inhibitor) reduced lipid droplets accumulation [127]. More recently, Dorn et al., generated iPSC-CMs from two ARVC patients, one harboring mutations in *PKP2* and the other one with mutations in the *MYH10* gene, and showed a novel mechanistic insight in the ARVC pathogenesis. Particularly, the authors demonstrated that cardiomyocyte identity is maintained by an active MRTF/SRF transcriptional program regulated by the RhoA-ROCK signaling pathway, which, in turn, is regulated by remodeling of the actin cytoskeleton at the intercalated disk. Genetic perturbation of this mechanosensory pathway activates an ectopic fat gene program during cardiomyocyte differentiation, which ultimately primes the cells to switch to a brown/beige adipocytic lineage when exposed to an adipogenic milieu. Additionally, they demonstrated, using in vivo fate mapping, that cardiac fat and a subset of cardiac muscle cells derive from a common precursor expressing Isl1 and Wt1, elegantly proposing a new and exciting mechanism responsible for fat accumulation in the heart of patients with ARVC [174].

### 7.5. Restrictive Cardiomyopathy

Restrictive cardiomyopathy (RCM) is a myocardial pathology causing an abnormal ventricular rigidity that impairs atrial and diastolic contraction, eventually leading to arrhythmias [175]. RCM may result from infiltrative diseases, such as sarcoidosis and amyloidosis [176], but is often associated with inherited or acquired mutations of sarcomere or cytoskeleton genes, such as *TNNI3* [177], *ACTC1* [178], *ACTN2* [179], *DES* [180], *MYBPC3* [181], and others [182,183]. Mutations on the *FLNC* gene, encoding the sarcomeric protein filamin C that is expressed in the contractile unit of cardiac and skeletal muscles, can also lead to RCM [184]. CMs differentiated from ESCs experimentally mutated to carry a missense variant of the *FLNC* gene showed impaired contractile activity [185]. A similar approach was used to introduce a *DES* mutation known to be responsible for RCM in an Iranian family, in iPSCs obtained from a healthy subject. The CMs derived from the mutated iPSCs highlighted the formation of cytoplasmic desmin aggregates that can account for the pathological phenotype [186].

### 7.6. Dilated Cardiomyopathy 

Dilated cardiomyopathy (DCM) is a group of heterogenous non-ischemic heart muscle diseases that associate with functional and structural abnormalities. Clinically, DCM features contractile dysfunction, left ventricular dilatation, and arrhythmias and frequently develops into heart failure. Non-ischemic cardiomyopathies are genetically quite heterogeneous [187]. Truncating *TTN* (encoding titin) mutations are the most prevalent for DCM development, accounting for about 30% of DCM patients [188]. Genes encoding for the sarcomeric β-myosin heavy chain (*MYH7*), the *LMNA* gene producing the nuclear lamina proteins lamin A and C, the desmin encoding gene (*DES*), ion channel coding genes (i.e., *SCN5A*), and many others were identified to be responsible for CDM, although at a lower prevalence compared to *TTN*. Gramlich et al., have developed an iPSC-based model system for DCM caused by truncating mutation in exon 326 of *TTN*. In their study, the authors demonstrated the beneficial effects of antisense oligonucleotides (AONs) exon skipping in the DCM phenotype. iPSC-CMs treated with AONs specific for exon 326 showed improved myofibril assembly and stability of the sarcomere. Using the same strategy, the authors were able to rescue the DCM phenotype in homozygous and heterozygous animals. Additionally, an association of a specific *TTN* mutation (Ser14450fsX4) with perturbations of the TK interacting Nbr1/p62/SQSTM1/MURF2 signalosome and a reduction of serum response factor (SRF)-dependent muscle gene expression was reported [189]. In another elegant study, the generation of iPSCs from patients with DCM was reported with the aim to uncover the mechanism underlying the disease. DCM-iPSC-CMs exhibited dystrophin deficiency, increased levels of cytosolic Ca^2+^, mitochondria damage, and apoptosis induced by CASP3 activation. Transcriptomic analyses of diseased iPSC-CMs and control iPSC-CMs allowed the molecular events underlying cardiac cell apoptosis as a consequence of mitochondria damage to be defined [190]. Wu and collaborators have profiled the β-adrenergic signaling during the differentiation and maturation of iPSC-CMs, demonstrating a novel epigenetic mechanism responsible for the compromised β-adrenergic signaling, able to induce both inotropic and chronotropic regulation of the contractile function in DCM-iPSC-CMs [191]. Yang et al., were able to recapitulate the contractile dysfunction typical of DCM using iPSC-CMs derived from patients with MYH7 E848G-induced systolic dysfunction and predicted that the restoration of the protein-protein interaction could be a proof-of-concept as a potential novel therapeutic strategy [192]. Lee at al. generated iPSC-CMs from three patients with distinct mutations (R225X, Q354, and T518fs) in the *LMNA* gene. Diseased CMs recapitulated the pathophysiological traits of *LMNA*-based CDM. Additionally, the authors showed the positive and protective effect of PTC124 application from nuclear abnormalities and apoptosis accompanied by improvements of contractile functions only in the *LMNA*^R225X^ mutant, while none of these effects were detected in the other two mutants [193]. In 2019, the production of the first iPSC-CMs model of DCM caused by p.S143P mutation in the *LMNA* gene was reported. In this model, diseased CMs displayed bradyarrhythmia and severe arrhythmic beats, beat rate variability, abnormal calcium handling, hypersensitivity to stress due to an increased expression of stress proteins, disorganized sarcomeres, and altered lamina structure upon hypoxia stress [128]. Exome sequencing and clinical studies have identified mutations in the B cell lymphoma 2–associated athanogene 3 (BAG3) gene as a potential cause of DCM [194]. BAG3 protein confers structural support in the cardiac cell by anchoring F-actin to α-actinin [195] and performs a quality control function by removal of mechanically damaged proteins such as filamin C from Z-discs via the chaperone-assisted selective autophagy pathway [196]. McDermott-Roe and co-workers have used genome-edited iPSC-derived cardiomyocytes to investigate the role of two mutations, R477H and complete loss of BAG3, in the molecular pathogenesis of DCMs. The study showed, in both genetic conditions, i.e., BAG3-RH variant and KO, a significant decline of fiber length and alignment, proteasome inhibition causing marked fiber disarray, and a weakening of the interaction between BAG3 and HSC/HSP70 in BAG3-RH. Moreover, cardiac fiber proteins underwent extensive mechanical stress and were continuously replaced [24]. More recently, an iPSC-CM model was used to characterize sarcomere microdomain interactions in the presence of the DCM-causing TNT- R173W mutation. In DCM-iPSC-derived CMs, the binding of troponin T (TnT) to tropomyosin was significantly reduced compared to isogenic control cells. The weak binding limits the ability of troponin to efficiently anchor on sarcomere filaments with a consequent destabilization of the sarcomere protein alignment. Additionally, diminished phosphorylation of troponin I (TnI) was detected as an effect of lower levels of PKA binding at sarcomeric microdomains in R173W mutated cardiomyocytes. R173W also accounts for an impaired interaction between sarcomere microdomain and cytoskeleton filaments via MYH7 and AMPK, leading to the disruption of sarcomere protein alignment and impaired contractility [197].

### 7.7. Left Ventricular Non-Compaction

Left ventricular non-compaction (LVNC) is a rare congenital heart disease with relevant potential complications including arrhythmias, heart failure, embolic events, trabeculations in the left ventricle, and dysfunction of the left ventricle (hypertrophy or dilation) [198]. The disease has been associated with the developmental defect of the myocardium. Genetic forms of LVNC are usually inherited as X-linked recessive or autosomal conditions and many genes (*TAZ*, *SCB5A*, *LMNA*, *MYH7*, *LDB3*) have been identified as causative of LVNC. Kodo and collaborators have generated iPSCs from LVCN patients harboring a mutation in the cardiac transcription factor *TBX20*. Diseased iPSC-derived CMs successfully recapitulated the pathological phenotypes of LVNC disease. The investigated molecular mechanism has been correlated to impaired transforming growth factor beta (TGFβ) signaling leading to decreased proliferation capability of LVNM-CMs. Mutations in *TBX20* alter the expression of TGFβ signaling modifiers such as PRDM16. By inhibition of the TGFβ signaling pathway and correction of the mutation in TBX20 using CRISPR-Cas9 technology, the authors were able to revert the disease phenotype [28].

### 7.8. Hypertrophic Cardiomyopathy

Hypertrophic cardiomyopathy (HCM) is a primary genetic myocardial disorder associated with left ventricular hypertrophy and/or hypertrophy of the septum, leading to a reduced cardiac output and sudden cardiac death, particularly in young adults. Histologically, HCM features hypertrophy of cardiac myocytes, disarray, and interstitial fibrosis. From a genetic point of view, HCM is associated with mutations occurring in genes encoding for sarcomeric proteins, among which *MYH7* (β-myosin heavy chain) and *MYBPC3* (myosin-binding protein C) account for about 50% of inherited HCM [199]. Other genes identified as responsible for the development of HCM but less common are *TNNC1*, *ACTC1*, *TPM1*, *TNNT2*, *TNNI3*, or *FHL1.* In very rare cases, mutations in genes encoding for Z-disc proteins such as *FLCN* or *ACTN2*, or genes involved in the homeostasis of calcium, like *PLN*, have also been shown to cause HCM. Mutations in mtDNA contribute to mitochondrial disorders for which cardiomyopathies are the most common clinical consequences. The molecular and pathological mechanisms underlying the HCM-associated m.2336T>C mutation localized in the mitochondrial 16S rRNA gene (MT-RNR2) were modelled using iPSC technology. iPSC-derived CMs exhibited the pathological features of hypertrophied cardiomyocytes with a significant reduction of 16S rRNA and its binding proteins. The authors reported impaired Ca^2+^ homeostasis due to mitochondrial dysfunction and a downstream induction of Ca^2+^-dependent cardiac hypertrophy [129]. Cohn and collaborators have engineered four human HCM models using CRISPR/Cas9 technology to generate isogenic mutations in *MYH7* and *MYBPC3* genes. Mutated CMs exhibited a state of hypercontractility due to increased tension and delayed relaxation kinetics. While the authors could not detect changes in the metabolism of diseased CMs under unstressed conditions, likely due to the persistence of a fetal metabolism in iPSC-CMs that favors glucose rather than fatty acids for energy production, they observed an increased in the p53 signaling pathway, suggesting that p53 ablation would be protective in HCM [200].

## 8. 3D Platforms for hiPSC-Cardiomyocytes-Based Cardiac Model Disease

Two-dimensional models are well-established and standardized methods widely used to study cardiovascular diseases; however, they cannot perfectly mimic and recapitulate the complexity of myocardium. On the other hand, in vivo animal models, such as rodents, do not fully recapitulate the human pathophysiology of cardiac diseases [201]. Three-dimensional (3D) cell culture systems have the capability to mimic the in vivo tissue microenvironment and architecture and to recapitulate cell-cell and cell-extracellular matrix (ECM) interactions; these features make this technology extremely appealing for tissue engineering, disease modeling, and drug discovery [202]. Spheroids, self-assembly three-dimensional cellular aggregates, represent the simplest 3D system. The most recent studies report the production of spheroids containing CMs co-cultivated with fibroblasts and endothelial cells to recapitulate the cardiac fibrosis phenotype [203] and for drug testing [204]. Cells can also be mixed with a scaffold matrix to create an engineered heart tissue (EHT). Hydrogel scaffold, composed of collagen, gelatine, hyaluronic acid, and extracts of native ECM, is the most used model to increase the maturity grade of iPSC-CMs or to measure tissue contraction [205]. Hydrogel can be substituted using artificial biomimetic materials, and the culture of CMs on a scaffold matrix can be supported by bioreactors and organ-on-chip and bioprinting technologies [206]. Three-dimensional platforms coupled with patient-derived iPSC-CMs has been successfully used to generate numerous cardiac disease models. Engineered iPSC-CM heart tissue (hEHT) has been used as a model to evaluate the abnormal contractile properties typical of HCM [207]. Using a heart-on-chip assay, Wang et al., created engineered myocardial tissue with iPSC-CMs carrying a mutation in the *TAZ* gene, responsible for the mitochondrial disorder known as Barth syndrome (BTHS). The 3D model showed that sarcomeres in BTHS are less organized compared to the control, leading to impaired contractile activity [25]. To model left ventricular hypertrophy (LVH), artificial human trabeculae were created by sticking CMs combined with hydrogel to two flexible wires into a microwell; the structures were conditioned using an electrical field to simulate the typical increased myocardial activity of an LVH subject, highlighting differences in mRNA expression and contractile activity compared to a healthy control [208]. These technologies are still in their infancy and still need in-depth study, but they certainly have great potential for drug discovery and personalized medicine, which are the two most relevant fields in cardiovascular research.

## 9. Conclusions

Cardiovascular diseases represent the leading cause of morbidity and death worldwide. Current therapies are mostly focused on relieving symptoms and preventing complications. Despite the progress in clinical research, many HF patients become refractory to standard and/or palliative medical therapies. Therefore, invasive cardiac transplantation remains the only choice for end-stage HF. Nevertheless, the procedure is highly risky and strikingly dependent on access to suitable donors. A comprehensive understanding of the molecular mechanisms underlying human cardiac diseases has been hampered by the lack of reliable model systems that mirror the human disease phenotype. ESCs have been considered the milestone for studying cardiac diseases since they can proliferate indefinitely and can give rise to any cell type, including cardiomyocytes. However, the destruction of the human embryo necessary for the derivation of ESCs has raised important ethical issues preventing and limiting their use. In addition, an ESC-based cardiac disease model cannot be considered totally reliable because of its misleading outcomes due to the individual-specific genetic and epigenetic background. The extraordinary advance accomplished in stem cell biology with the discovery of human iPSCs has completely reshaped our approach to studying human diseases. iPSC technology, through the generation of specific cellular models carrying pathogenetic mutations responsible for the disease phenotype, has allowed novel molecular targets and signaling pathways to be uncovered for the development of new therapeutic strategies. Outstanding advances in differentiation methods, in combination with new impressive genome editing tools like CRISPR-Cas9, have allowed the generation of patient-specific CMs models and their respective isogenic controls. Although iPSCs have generated great enthusiasm within the scientific community, concerns have been raised regarding their real equivalence to ESCs, but little conclusive evidence has been reported regarding iPSC and ESC cardiac derivatives, strengthening the applications of iPSCs in basic and clinical cardiac research. Concerns around iPSC technology are not just about their similarity to ESCs, but also concern the intrinsic properties of iPSC-derived cardiomyocytes. (1) iPSC-CMs typically exhibit immature structural and functional properties resembling phenotypically and functionally fetal cardiomyocytes: while this feature may even be advantageous to model early disease stages, particularly relevant for diseases showing early onset, the low maturity may create problems with regard to the use of iPSC-CMs in clinically relevant settings such as novel drug testing or evaluating their efficacy/or toxicity; moreover, the relatively immature phenotype of iPSC-CMs may mask important pathological mechanisms typical of adult-onset cardiac diseases; (2) iPSC-CMs present batch wise variations in differentiation; (3) major limitations of iPSC technology are associated with the reprogramming process: reprogrammed cells might retain the epigenetic signature of the somatic cell from which they were derived, chromosomal aberrations and/or accumulation of mutations, and genomic instability. Therefore, international standard processes are required to characterize and validate these cells at every stage, with special attention to their use in clinics to ensure safety. Despite the mentioned limitations, the power of iPSC technology for clinical and basic cardiovascular research remains undisputed.

## Abbreviations

CVD	(Cardiovascular Disease)
iPSCs	(Induced Pluripotent Stem Cells)
HF	(Heart Failure)
I/R	(Ischemic/Reperfusion)
MI	(Myocardial Infarction)
hPSCs	(Human Pluripotent Stem Cells)
ESCs	(Embryonic Stem Cells)
CMs	(Cardiomyocytes)
iPSC-CMs	(Induced Pluripotent Stem Cells-derived Cardiomyocytes)
ICM	(Inner Cell Mass)
ZNFs	(Zinc-Finger Nucleases)
TALENs	(Transcription-Activator Like Effector Nucleases)
CRISPR-Cas9	(Clustered Regularly Interspaced Short Palindromic Repeats-associated protein 9)
DSBs	(Double-Strand DNA Breaks)
NHEJ	(Nonhomologous End Joining)
HR	(Homologous Recombination)
DCM	(Dilated Cardiomyopathy)
BTHS	(Barth Syndrome)
LQTS	(Long QT Syndrome)
BS	(Brugada Syndrome)
LVNC	(Left Ventricular Non-Compaction)
END-2	(Endoderm-like cells)
EB	(Embryod Body)
BMPs	(Bone Morphogenetic Proteins)
CPCs	(Cardiac Progenitor Cells)
SMCs	(Smooth Muscle Cells)
CAR-T	(Chimeric Antigen Receptor T cell)
GWAS	(Genome-Wide Association Studies)
NGS	(Next Generation Sequencing)
ALS	(Amyotrophic Lateral Sclerosis)
TKIs	(Twenty-one Tyrosine Kinase Inhibitors)
VEGF	(Vascular Endothelial Growth Factor)
PDGF	(Platelet-Derived Growth Factor)
HLA	(Human Leukocyte Antigen)
